# Understanding the complex chromatin dynamics in primary human neutrophils during PMA-induced NET formation

**DOI:** 10.3389/fimmu.2024.1445638

**Published:** 2024-10-25

**Authors:** Brandi Atteberry, Benjamin P. Berman, Theresa K. Kelly, Justin Cayford

**Affiliations:** ^1^ Innovation Laboratory, Volition America, Carlsbad, CA, United States; ^2^ Department of Developmental Biology and Cancer Research, The Hebrew University of Jerusalem, Jerusalem, Israel

**Keywords:** NETosis, neutrophil, ATAC-seq, chromatin, PMA, sepsis, innate immune system, NET formation

## Abstract

**Background:**

Primary human neutrophils play a pivotal role in innate immunity, mainly through the formation of neutrophil extracellular traps (NETs) in a process known as NETosis. This cell-death pathway is crucial for combating infections but is also implicated in many inflammatory diseases, such as sepsis, systemic lupus erythematosus, and rheumatoid arthritis.

**Methods:**

The study presented here investigates chromatin dynamics during NET formation by stimulating primary human neutrophils with phorbol 12-myristate 13-acetate (PMA). We adapt the ATAC-Seq (assay for transposase-accessible chromatin using sequencing) method to isolated neutrophils and characterize a time-dependent chromatin response.

**Results:**

We found that chromatin accessibility patterns are consistent across individual donors and most chromatin changes occur within 30 min, with many continuing across the 90 min assessed in this study. Regulatory regions gaining accessibility were associated with the activity of pathways that have been implicated in NOX-dependent NET formation.

**Conclusions:**

Our findings increase the understanding of the chromatin changes underlying NET formation and also identify potential early-acting targets for modulating this process in inflammatory diseases.

## Introduction

Neutrophils are the most abundant white blood cell type in humans and play crucial roles in the innate immune system as one of the first lines of defense against infection (1). In response to pathogens, neutrophils undergo a process called NETosis, which culminates in the release of neutrophil extracellular traps (NETs) composed of chromatin fibers adorned with anti-microbial proteins and proteases that entrap and neutralize a variety of microbial invaders, including bacteria, viruses, and fungi (2–6). Although NET formation represents a crucial defense mechanism, dysregulation of this process contributes to the pathogenesis of diverse inflammatory conditions, including sepsis, systemic lupus erythematosus (SLE), and rheumatoid arthritis (RA) (6). This underscores the delicate balance required for effective immune responses, wherein the precise regulation of NET formation is vital in maintaining immune homeostasis and preventing inflammatory disease progression (7–9). In sepsis, excessive NET formation can cause tissue damage and organ dysfunction; in SLE, neutrophils and aberrant NET formation could be a cause of an increase in anti-dsDNA antibodies; and in RA, aberrant NET formation perpetuates chronic inflammation and joint tissue destruction (10–13). Thus, maintaining the delicate balance of regulating NET formation is crucial for effective immune responses and preventing inflammatory diseases.

Understanding the mechanisms underlying NET formation is critical for identifying biomarkers and developing therapeutic interventions. However, as neutrophils are inherently designed to sense environmental perturbations, they are highly sensitive to external stimuli. Additionally, they have short half-lives and limited survival both *in vitro* and *in vivo*, which complicates their study (14). Their viability is further compromised by freezing, making them challenging to work with experimentally. Although neutrophil-like cell lines such as differentiated HL-60 cells have been employed to investigate the regulation of NET formation, these models do not faithfully replicate the behavior of primary neutrophils (15). Moreover, the study of isolated neutrophils fails to capture the complexity of cell-cell interactions and indirect signaling among various immune cell types. Furthermore, findings based on studying NET formation in murine models do not always translate to human biology (16, 17). Investigating NET formation within the context of human primary neutrophils is crucial for translating laboratory-based findings into clinical advancements.

NET formation has been shown to occur via two distinct pathways: dependent or independent of NADPH oxidase (NOX) (1, 18). Phorbol 12-myristate 13-acetate (PMA) stimulation has been shown to be NOX dependent (19), whereas other activators such as calcium ionophore are NOX independent (20). Both the NOX-dependent and -independent NET formation pathways likely contribute to host defense against pathogens, and dysregulation of either pathway can exacerbate inflammatory conditions. For instance, excessive reactive oxygen species (ROS) production in the NOX pathway can lead to tissue damage and inflammation, and the aberrant activation of protein kinases in the non-oxidative pathway can contribute to autoimmune responses and tissue injury (21). Understanding the intricate regulation of these pathways is crucial for developing targeted therapeutic strategies to modulate NET formation and mitigate inflammatory diseases.

Chromatin decondensation studies have investigated the dynamics of chromatin modifications during NET formation, highlighting the importance of histone citrullination by PAD4 in chromatin decondensation and trap formation (22, 23). Other research has explored the impact of chromatin regulators on NET release (7). In this study, we aimed to probe the temporal dynamics of chromatin reorganization during PMA activation of NET formation (18). Leveraging the assay for transposase-accessible chromatin using sequencing (ATAC-Seq) (24) with the incorporation of fixation methods (25), we interrogated the landscape of chromatin accessibility at distinct timepoints following PMA stimulation. By outlining the spatial and temporal trends in chromatin remodeling, we sought to unravel the underlying regulatory networks orchestrating NET formation and identify candidate epigenetic modifiers that may provide answers to therapeutic intervention. We found that chromatin accessibility patterns are consistent across individual donors and that chromatin dynamics were organized across the genome. Finally, the chromatin accessibility patterns highlight key regulatory mechanisms activated during PMA-stimulated NET formation.

## Materials and methods

### Tn5 assembly

Recombinant Tn5 transposase protein (Active Motif #81284) lots were assembled with IDT custom oligos mosaic end (ME) ME_Rev, ME_A, and ME_B, and activity was tested as described previously (24) before initiating ATAC-seq experiments.

### Whole-blood collection and neutrophil isolation

Whole blood was obtained from healthy donors (**Supplementary Table 1**) in K2-EDTA tubes (BD #366643) (PrecisionMed, San Diego, CA, USA). The research was approved under WCG IRB Protocol number 20181025 and all human participants provided written informed consent. Each subject was self-declared healthy, between the ages of 18–50, with a BMI of <30, and not taking NSAIDs. Neutrophil isolation was initiated within 1 h of blood collection using an MACSxpress Whole Blood Neutrophil Isolation Kit for humans (Miltenyl Biotec #130-104-434). Briefly, a fraction of the prepared bead mixture was added to the blood sample, followed by incubation on a rotator for cell binding. Subsequent magnetic separation yielded isolated neutrophils, which were washed in 1× PBS and red blood cell lysis buffer to eliminate contaminants, resulting in >97% pure neutrophils (26) with >98% survivability as measured by Trypan Blue staining on a Countess 3 Cell Counter (Thermo Fisher Scientific). The resulting cell pellet was resuspended in prewarmed Roswell Park Memorial Institute (RPMI) at 37°C for induction or untreated conditions.

### NET imaging and immunocytochemistry

Isolated neutrophils were stimulated with 100 nM PMA and imaged using the S3 Live-Cell Analysis System (Sartorious) as described previously (26). For immunocytochemistry, acid-washed 12 mm coverslips (Fisher Scientific, 12-541-026) were placed in a 35 mm dish (Thermo Fisher Scientific, 171099) and coated with 70 µl of 1 mg/ml fibronectin (Sigma-Aldrich, F1141) at 37°C for 1 h. After aspirating fibronectin, the dish was seeded with 2 ml of human neutrophils (20,000 cells/ml). Neutrophils were stimulated with PMA (100 nM) or DMSO (0.1%) for 3 hours at 37°C. Cells were fixed with 4% paraformaldehyde for 10 min and permeabilized with 0.1% Triton X-100 in PBS for 5 min. Coverslips were blocked with 5% donkey serum in PBS + 0.1% Tween 20 overnight at 4°C. After PBS washes, primary antibodies (50 µl) (**Supplementary Table 2**) were added and incubated overnight at 4°C. Secondary antibodies (**Supplementary Table 2**) were added after washes and incubated at room temperature for 1 h. Coverslips were stained with DAPI and Cell Mask Orange (Thermo Fisher Scientific, C10045) in PBS for 10 min, washed, rinsed in DI water, and mounted with Fluoromount (Sigma-Aldrich, F4680). Imaging was performed at the Nikon Imaging Center at the University of California, San Diego, using an AXR confocal microscope.

### Induction with a PMA/DMSO time course

Isolated neutrophils were resuspended in prewarmed (37°C) RPMI 1640 Medium (Gibco Cat #11875135) at a concentration of 1–2 × 10^6^ cells/ml in a final volume of 10 ml and treated with either 100 nM of PMA (Sigma-Aldrich, Cat #P1585) or an equivalent volume of DMSO (ATCC Cat #4-X) for each timepoint. Subsequently, each tube was incubated at 37°C with 5% CO_2_, and 1 ml aliquots were collected prior to treatment and at 30, 60, and 90 min intervals in duplicate. Each aliquot was fixed as described below for further processing.

### Fixation

A 10× solution of formaldehyde (formaldehyde 11%, NaCl 1M, EDTA 0.1 mM, and HEPES 0.5 mM) was prepared, and a 1:10 (1% final) volume was added to isolated neutrophils for fixation. After incubation at room temperature for 10 min, fixation was quenched with a 1:20 volume of glycine (2.5 M), followed by incubation on ice for 5 min and then centrifugation at 300 × *g*. Cells were subsequently washed in ice-cold 1× PBS; cells were then pelleted again to retain only intact neutrophils and wash away any potential NET fragments or other DNA. The cells were counted and 125,000 cells per condition/timepoint/replicate were flash frozen in liquid nitrogen and stored at –80°C until further processing.

### ATAC-Seq

Unfixed ATAC-Seq was performed as previously reported without major changes (27). The fixed ATAC-Seq protocol was adapted from Buenrostro et al. utilizing information from Chen et al. to determine fixation steps (25, 27), in which each frozen cell pellet (125,000 cells) was resuspended in 50 µl of ATAC RSB Complete [10 mM Tris (pH 7.5), 3 mM MgCl_2_, 10 mM NaCl, 0.1% NP-40, 0.1% Tween 20, and 0.01% digitonin] and incubated on ice for 3 min. An aliquot of 1 m of cold ATAC RSB NULL [10 mM Tris (pH 7.5), 3 mM MgCl_2_, 10 mM NaCl, and 0.1% Tween 20] was added, and the mixture was inverted three times before pelleting nuclei at 500 × *g* for 10 min at 4°C. Supernatant was removed and the pellet was resuspended in Omni-ATAC Mix (28) {1×TD [10 mM Tris (pH 7.5), 5 mM MgCl_2_, 10% dimethylformamide], 1× PBS, 0.01% digitonin, and 0.01% Tween 20} and assembled Tn5 (3.26 µM) was added to a final volume of 50 µl and incubated at 37°C for 30 min in a thermal cycler and the reaction was stopped by incubating on ice for at least 5 min. ATAC-Seq reverse cross-link buffer [50 mM Tris (pH 8.0), 200 mM NaCl, 1 mM EDTA, and 1% SDS] and proteinase K (1.25 µl) (Invitrogen, #25530049) were subsequently added and incubated at 65°C overnight, and DNA was isolated using Zymo DNA purification (Zymo, #D4013) and eluted in 18.5 µl of pre-warmed 10 mM Tris (pH 8.0).

### DNA library preparation

DNA libraries were generated using NEB Next Ultra II Q5 Master Mix (NEB# M0544L) and amplified by PCR as described previously (27). PCR amplicons were purified by size selection (0.5× followed by 1.2×) using AMPure XP Reagent (Beckman Coulter, #A63881), quantified using a Qubit Flex Fluorometer (Thermo Fisher Scientific, #Q3327), and analyzed using an Agilent TapeStation 4150 with Agilent High Sensitivity D1000 tapes (Agilent, #5067-5594). Libraries were quantified using a Roche KAPA Library Quantification Kit (Roche, #0796014000) and pooled for sequencing on an Illumina NextSeq2500 (**Supplementary Table 3**).

### ATAC-Seq processing and peak calling

ATAC-Seq alignment and processing were conducted using an ATAC-Seq Nextflow pipeline (https://nf-co.re/atacseq/2.1.2) (29) with the nf-core framework (30); default settings were used unless otherwise specified. Samples were aligned to the hg38 reference genome with a fragment size parameter set to 200. Peak calling was performed using MACS2 (31), with narrow peaks identified at a false discovery rate (FDR) of 0.01 (macs_fdr=0.01). We followed the general ENCODE current standards for sequencing (32).

### Untreated ATAC-Seq analysis

Consensus peaks (*.featureCounts.txt*)* and annotated peaks (*.annotatePeaks.txt) were generated using the nf-core pipeline and utilized for subsequent analysis. To normalize read counts across samples, scaling factors from bigwig normalization (*./bigwig/scale) (https://cran.r-project.org/web/packages/scales/index.html) were applied to balance the total number of reads across samples. For untreated samples, normalized peak counts were combined with annotated peaks to create a consensus peak dataset. UpSetR (https://cran.r-project.org/web/packages/UpSetR/index.html) plots were generated using the *.boolean.annotatePeaks.txt files. Additional metrics, including read counts, peak annotations, bigwigs, fraction of reads in peaks (FRiP) scores, insert sizes, and other alignment metrics, were generated using the nf-core ATAC-Seq pipeline.

### Treated ATAC-Seq analysis

Treated samples were analyzed similarly to untreated samples with some modifications. Samples were normalized in the same manner as above and then divided into six groups based on the time course (T30, T60, and T90 min) and treatment (DMSO vs. PMA). To retain the most relevant peaks, we filtered the peaks using the *.boolean.annotatePeaks.txt files. Peaks were retained if at least two-thirds of the donors had a peak within that interval. The peaks were then concatenated, retaining only the unique intervals. The normalized counts for these consensus peaks were analyzed using DESeq2 (33). The DESeqDataSetFromMatrix() function was used to create the dataset, followed by the assay() function for data extraction. Principal component analysis (PCA) scores were calculated using prcomp, and PCA contributions were calculated with the summary() command. Pairwise comparisons were performed with DESeq(), and results were summarized using the results() function. Significant regions (padj < 0.01) were z-score normalized based on the normalized peak counts, and heatmaps were generated using pheatmap (https://cran.r-project.org/web/packages/pheatmap/index.html) with row clustering. Volcano plots were created from the results() function from DESeq2, with significant regions defined by -log_10_(padj) >20. For all-vs-all comparisons, significant regions from each grouping (treatment and timepoint) with padj <0.01 were included. Pheatmap was then used with both row and column clustering. To analyze overlaps between DMSO Early vs. PMA Early, Mid, and Late, an inner_join (tidyverse) (34) was used, followed by eulerr version 7.0.2 (https://CRAN.R-project.org/package=eulerr) for venn diagram visualization in a custom R script.

### HOMER analysis

To determine whether there was any enrichment of motifs in the gained or lost peaks after PMA stimulation, HOMER (35) analysis was completed on the top 1,000 significant regions based on Wald scoring for all PMA or DMSO treatments (Early, Mid, and Late) using findMotifsGenome.pl with hg38 and standard settings.

## Results

### The chromatin structure of primary human neutrophils is organized and stable with common accessibility patterns across healthy donors

**Figure 1:**
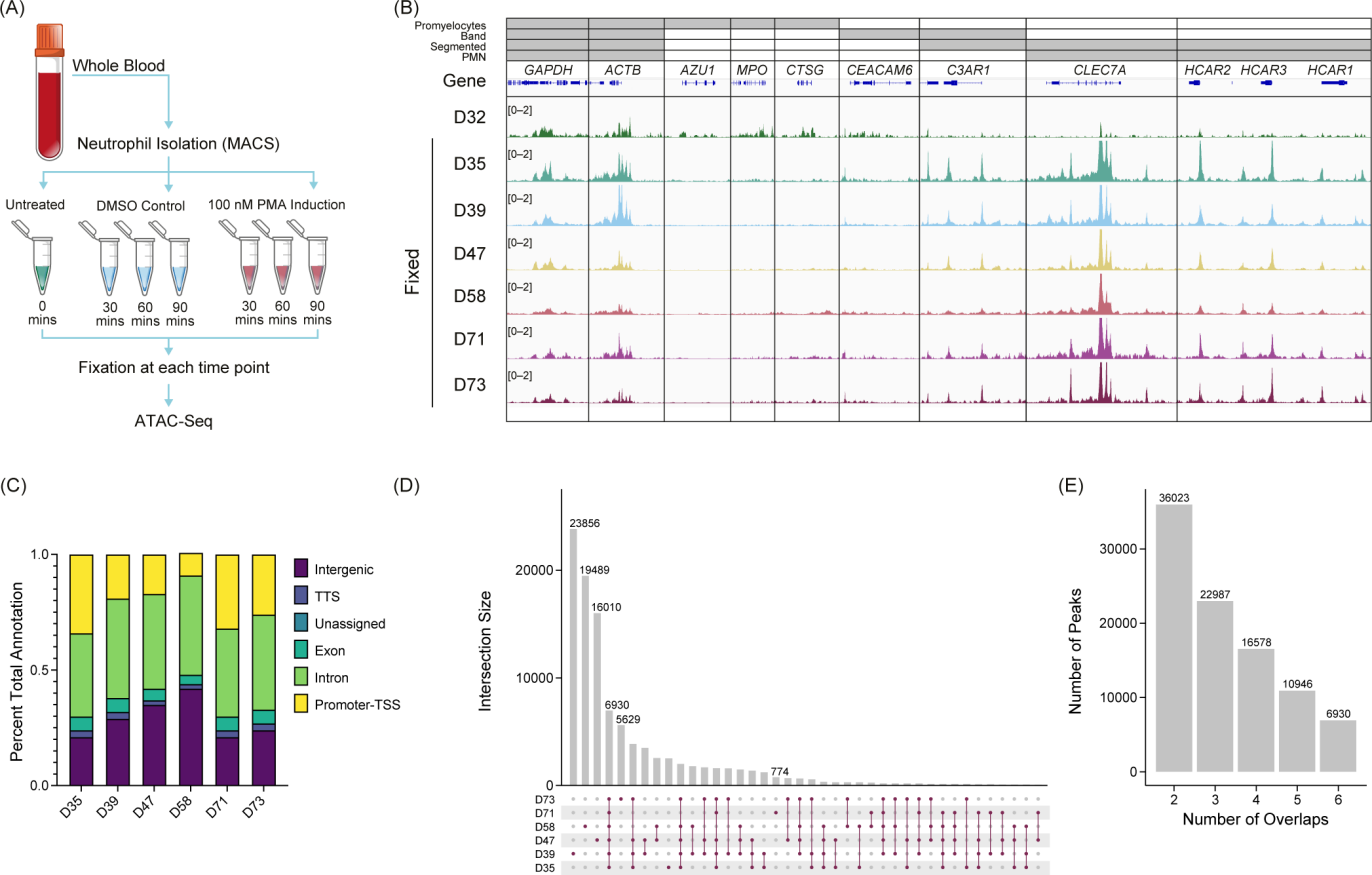
Untreated primary human neutrophil ATAC-Seq shows consistent peaks. **(A)** Experimental schematic. Fresh whole blood was collected, and neutrophils were isolated using an MACSxpress Whole Blood Neutrophil Isolation Kit for humans (Miltenyi Biotec #130-104-434). Neutrophils were then stimulated with phorbol 12-myristate 13-acetate (PMA) or dimethyl sulfoxide (DMSO) for a given time, fixed with formaldehyde, and then used for ATAC-Seq. **(B)** Merged replicate tracks visualized using the IGV genome browser with untreated healthy donors (n=7), standard ATAC-Seq (D32, top), and fixed samples (D35, D39, D47, D58, D71, and D72) are shown below. The top bars (gray) indicate the stage of neutrophil development at which H3K4me3 signal was present [promyelocytes, band neutrophils (band), segmented, or polymorphonuclear neutrophils (PMNs)]. Housekeeping genes (*GAPDH* and *ACTB*) are shown, followed by several other neutrophil genes (*AZU1*, *MPO*, *CTSG*, *CEACAM6*, *C3AR1*, *CLEC7A*, and *HCAR1*, *HCAR2*, and *HCAR3*). **(C)** Peak annotations generated on called MACS2 peaks (q <0.01) for the fixed donors shown as a percentage. TSS, transcription start site. TTS, transcription termination site. **(D)** UpSetR plot for the fixed donors showing the intersections of untreated neutrophils. **(E)** Total number of consensus peaks (y-axis) based on the overlap for the indicated number of donors (x-axis) for the fixed samples (n=6).

To gain a deeper understanding of the chromatin structure of neutrophils, we performed ATAC-Seq with formaldehyde fixation of unstimulated (n=6), PMA-, or DMSO-stimulated (n=3) primary human neutrophils (**Figure 1A**), and standard (unfixed) ATAC-Seq in one donor (**Figure 1B** – D32). To assess the open chromatin regions across donors, we looked at genes associated with the activating H3K4me3 modification across various stages of neutrophil development (36) (**Figure 1B**). We found the expected overlap between identified H3K4me3 regions and ATAC-Seq peaks in our samples and what has been reported for polymorphonuclear neutrophils (PMN) (37), indicating that there is stable organization of accessible chromatin in neutrophils, across donors. Fixed ATAC-Seq samples from donors (D35, D39, D47, D58, D71, and D73) had a higher signal overall than the unfixed sample (D32), as highlighted in the *CLEC7A* and *HCAR* loci (**Figure 1B**). There was also signal in those loci in D32 but the signal to noise was much lower. D32 also had signal present in regions such as *AZU1*, *MPO*, and *CTSG*, which are more associated with promyelocytes and were not represented in the fixed samples (**Figure 1B**).

We identified peaks using MACS2 (q <0.01) and found that accessibility regions were present across a variety of genomic features with some variability across samples (**Figure 1C**), which potentially was driven by donor-to-donor differences. We found the peaks were reproducible across the six healthy donors indicated by a high number of consensus peaks. Donors D39, D47, and D58 had a high number of unique peaks (**Figure 1D**), which may be due to the variable sequencing depth among samples (**Supplementary Figure 1A**). There was an overall trend of increased FRiP as the number of peaks increased with some variability, which is not unusual when using primary cells (**Supplementary Figure 1B**). The number of peaks shared across all six fixed donors was 6,930 (**Figure 1E**). The unfixed sample, D32, had significantly more peaks than the fixed samples (>100,000) and most of the peaks did not correlate with the fixed samples. Of the approximate 7,000 peaks found in all fixed samples, over 81% were also shared with the unfixed donor (**Supplementary Figure 1C**). To generate a consensus neutrophil peak set across the donors, we set the threshold of the peak being present in at least half of the fixed samples for the peak to be included as a consensus peak. We found that approximately 23,000 peaks were present using this threshold (**Figure 1E**). These findings suggest a well-structured and regulated chromatin landscape of primary human neutrophils.

### Chromatin dynamics occur throughout PMA stimulation compared with DMSO controls

To assess chromatin changes associated with PMA-induced NET formation, we treated neutrophils from three donors with PMA or DMSO, collected samples at 30, 60, and 90 min, and subsequently ran ATAC-Seq (**Supplementary Figure 2A**). These timepoints preceded NET release; therefore, to confirm that these conditions resulted in NET formation we performed live-cell imaging on parallel samples using an extended time course and Cytotox dye intercalation and showed that DNA release began at 2 h (**Supplementary Figure 3A**) and that NETs contained markers associated with NET formation, such as H3.1 (**Supplementary Figure 3B**), H3R8cit (**Supplementary Figure 3C**), myeloperoxidase (MPO) (**Supplementary Figure 3D**), neutrophil elastase (NE) (**Supplementary Figure 3E**), and nucleosome (**Supplementary Figure 3F**).

**Figure 2:**
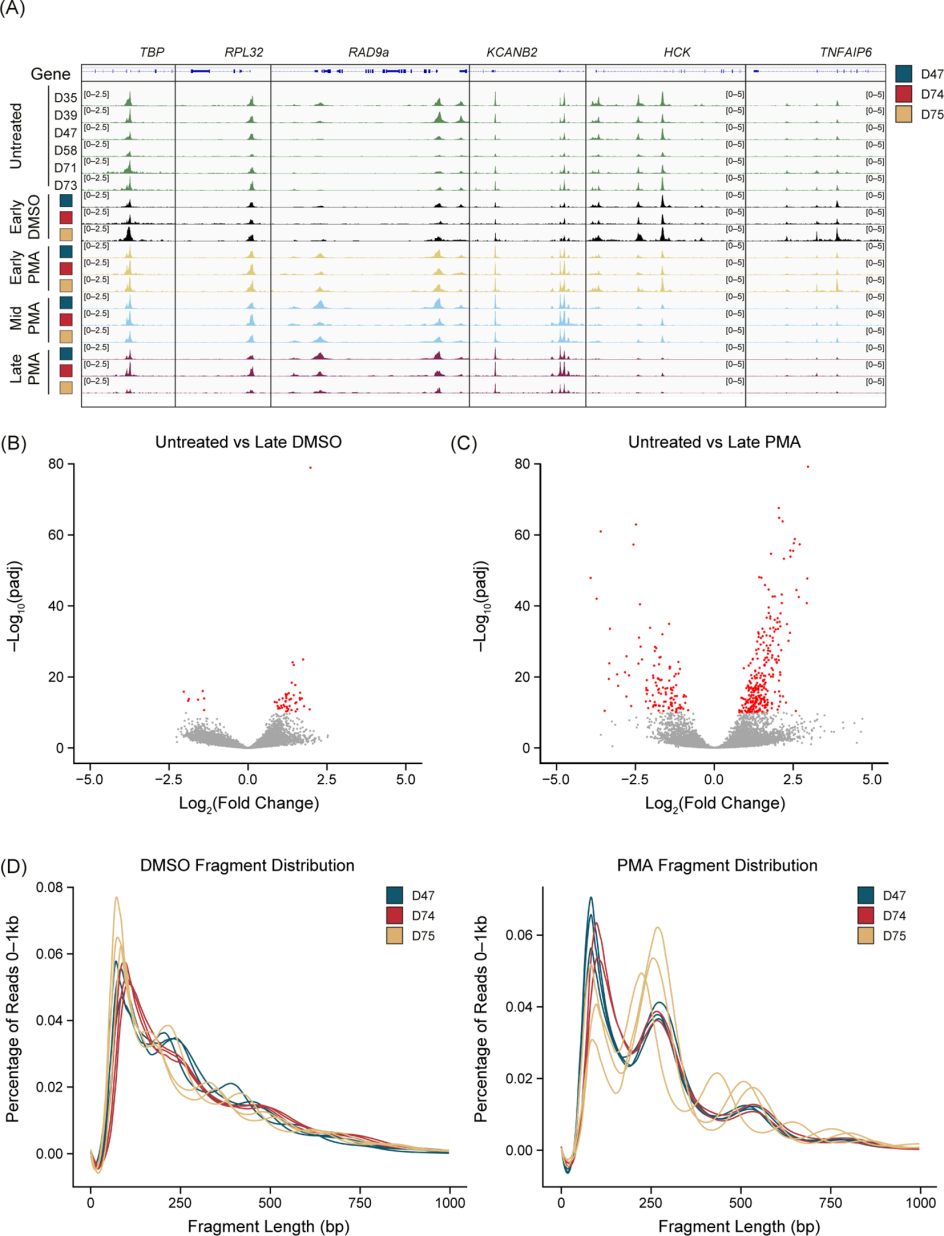
PMA generates unique chromatin dynamics compared with DMSO or untreated neutrophils. **(A)** Merged replicate tracks visualized using the IGV genome browser with untreated healthy donors (n=6, green tracks) and phorbol 12-myristate 13-acetate (PMA)- or dimethyl sulfoxide (DMSO)-treated donors (n=3) at Early (T30 minutes – yellow tracks), Mid (T60 minutes – blue tracks), and Late (T90 minutes – purple tracks). The treated donors D47 (blue), D74 (red), and D75 (gold) are shown at known housekeeping genes: *TBP* and *RPL32*. Examples of gained peaks are shown at the *RAD9a* and *KCANB2* loci. Decreased peak examples are shown at the *HCK* and *TNFAIP6* loci. **(B)** Volcano plot comparing untreated samples with Late DMSO. DESeq2 was used for a pairwise comparison and then plotted. The -log_10_(p.adj) value is graphed on the y-axis and significant values are plotted in red [-log_10_(p.adj) <10]. Log_2_(fold change) is indicated on the x-axis. **(C)** Similar to **Figure 2B** but comparing untreated samples vs. Late PMA. **(D)** Sequencing fragment distribution for the DMSO controls (left) and PMA samples (right) plotted as base pairs (bp) on the x-axis from 0–1,000, grouped into 20 bp windows. The percentage of total reads over 0–1,000 is plotted on the y-axis. Donors are indicated by color (D47, blue; D74, red; D75, gold). Timepoints (Early, Mid, and Late) are graphed per donor.

There was a reproducible chromatin response to PMA stimulation but not with a DMSO vehicle control compared with the untreated samples (**Figure 2A**). We found consistent chromatin accessibility in the housekeeping genes *TBP*, *RPL32* (**Figure 2A**), *GAPDH*, and *ACTB* (data not shown) throughout the untreated, DMSO, and PMA treatments. Interestingly, peaks were both gained or lost upon PMA stimulation. A gain of peaks generally appeared within 30 min (Early) of stimulation, as highlighted at the *RAD9a* and *KCANB2* loci (**Figure 2A**). Loss of peaks started to appear at the Early timepoint but were not fully resolved until 60–90 min (Mid-Late). The *HCK* and *TNFAIP6* loci are two examples that showed this reduction of ATAC-Seq signal over the time course reflective the general genome-wide trend (**Figure 2A**).

We found that DMSO-treated samples had a low number of differential accessible regions (DARs) at the most extreme pairwise comparison (DESeq2) of untreated versus Late DMSO (90 min incubation) (**Figure 2B**). However, there were many DARs between the untreated and the Late PMA treatment across the 3 donors (**Figure 2C**). Volcano plots showed a much greater response overall in the PMA comparison in both the *p*-values present and the fold change. As a result, we used Early-DMSO-treated samples as the analysis control.

Interestingly, we observed a shift toward mono- and di-nucleosome fragments after PMA treatment at all timepoints (**Figure 2D**), indicating a dynamic chromatin response. Despite the nucleosome pattern, PMA stimulation did not have an impact on peak localization (**Supplementary Figure 2B**) as there was a larger impact from donors than from the stimulation condition or timepoint. The number of unique peaks varied throughout the time course for all donors (**Supplementary Figure 2C**). To minimize the impact of peak number at a specific timepoint and treatment, a peak was only retained if it was present in two of the three donors and was used in subsequent analyses.

### PMA induction of NET formation leads to an organized chromatin response across time and donors

**Figure 3:**
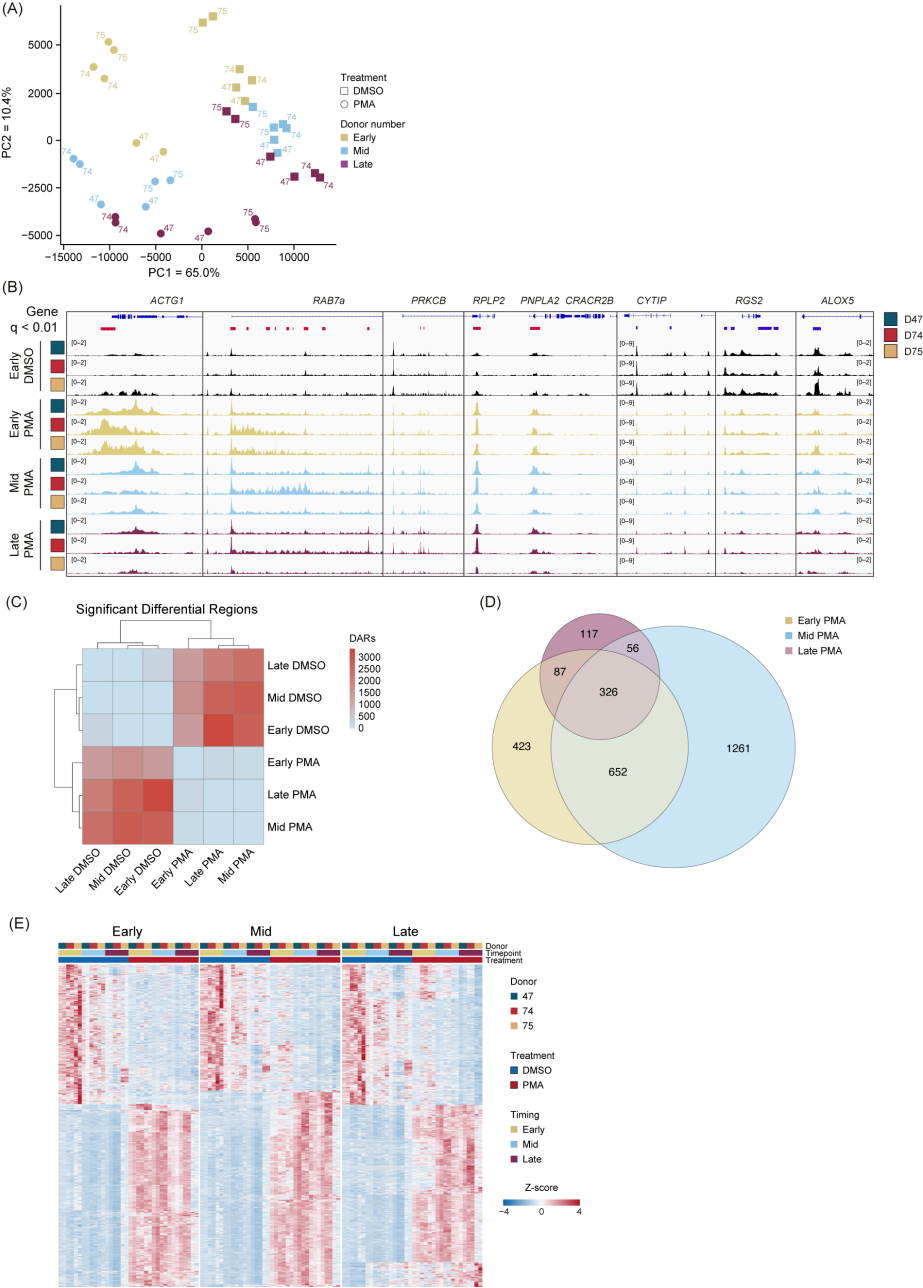
PMA stimulation causes a genome-wide alteration of chromatin accessibility across donors and time. **(A)** Principal component analysis (PCA) based on unbiased clustering of dimethyl sulfoxide (DMSO) vs. phorbol 12-myristate 13-acetate (PMA) using the unmerged data. Timepoints are indicated by color (Early, gold; Mid, blue; Late, purple). Treatment is indicated by squares (DMSO) or circles (PMA). PC1 (x-axis) represented 65% of the total variance and PC2 (y-axis) represented 10.4% of variance. Donors are indicated by the number adjacent to the data point. **(B)** Merged replicate tracks visualized with the IGV genome browser with Early DMSO (black), Early PMA (gold), Mid PMA (blue), and Late PMA (purple) at various gene loci. Donors are indicated by color (D47, blue; D74, red; D75, gold). Significant differential accessible regions based on Early DMSO vs. each timepoint [DESeq2 p.adj >0.01 and log2(fold change) less than −1.5 or greater than 1.5] are shown below the genes, where gained peaks are shown in red and loss of peaks are shown in blue. The bimodal response is shown at *ACTG1* and *RAB7A*, increased accessibility at *PRKCB*, *RPLP2*, and *PNPLA2*, and decreased accessibility at *CYTIP*, *RGS2*, and *ALOX5*. **(C)** Heatmap showing the number of differential accessible regions (DARs) between all pairwise comparisons (each timepoint for each treatment condition) [DESeq2 p.adj >0.01 and log2(fold change) less than −1.5 or greater than 1.5]. **(D)** Venn diagram of the overlaps between Early DMSO and the PMA treatments (Early, gold; Mid, blue; Late, purple). The total number of DARs in each condition is indicated. **(E)** Z-score normalized heatmap representation of the Early DMSO vs. PMA treatments (Early, left; Mid, middle; Late, right). Donors are indicated by color (D47, blue; D74, red; D75, gold). Timepoints (Early, Mid, and Late) are graphed per donor. Treatment is indicated by DMSO (blue) and PMA (red). The top 1,000 DARs were selected based on the Wald score. Columns are organized by treatment, timepoint, and donor across all conditions, and rows are hierarchically clustered.

To determine whether PMA stimulation responses were unique compared with DMSO, we performed unsupervised clustering PCA comparing PMA with DMSO using unmerged replicates (**Figure 3A**). The first principal component (PC1) accounted for 65% of the proportion of variance between PMA treatment and DMSO vehicle controls, and PC2 accounted for 10.4% of the variance and separated each group based on time (Early, Mid, and Late). Importantly, we did not observe any obvious donor batch effects (**Figure 3A**).

There are several examples of chromatin accessibility changes in inflammatory genes or genes known to be important in neutrophil functions (**Figure 3B**), with a few different modes of chromatin accessible changes observed. Regions, such as *ACTG1* and *RAB7a*, were more accessible in the Early and into the Mid stage, followed by less accessible at the Late timepoint. We found some instances of increased accessibility at intragenic (PRKCB) or transcription start sites (RPLP2 and PNPLA2). Additionally, there were regions that had a reduction in peaks (*CYTIP*, *RGS2*, and *ALOX5*) (**Figure 3B**). These represent the dynamic and structured chromatin response seen genome-wide during PMA activation across donors.

We next performed pairwise comparisons (DESeq2) between every grouping (each timepoint and treatment) and calculated the total number of differentially accessible regions (DARs) (q <0.01). The DARs highlighted large differences between the PMA and DMSO groupings but very minor differences within treatment groups (**Figure 3C**). Owing to limited variability in the DMSO treatment group, future comparisons used only the Early DMSO as the control. Overlapping DARs between the PMA groups (Early, Mid, and Late) were identified using a q-value <0.01 and a log_2_ fold change greater than 1.5 or less than −1.5 (**Figure 3D**). Almost 40% (1,121/2,922) of the DARs were sustained in at least two of the timepoints, with the majority of regions being found in the Early or Mid timepoints. These data suggest an initial response at 30 min, followed by a secondary response at 60 min. After 60 min, there were less than 5% (117/2,922) unique changes (**Figure 3D**). Next, pairwise comparisons of the Early DMSO vs. each PMA treatment time were made. The top 1,000 DARs were z-score normalized and plotted as a heatmap (**Figure 3E**). The results suggest the stability of the DARs over time and across donors over each comparison.

### PMA induction has unique early and mid chromatin responses that are associated with transcription factors associated with NET formation with limited changes after 60 minutes

**Figure 4:**
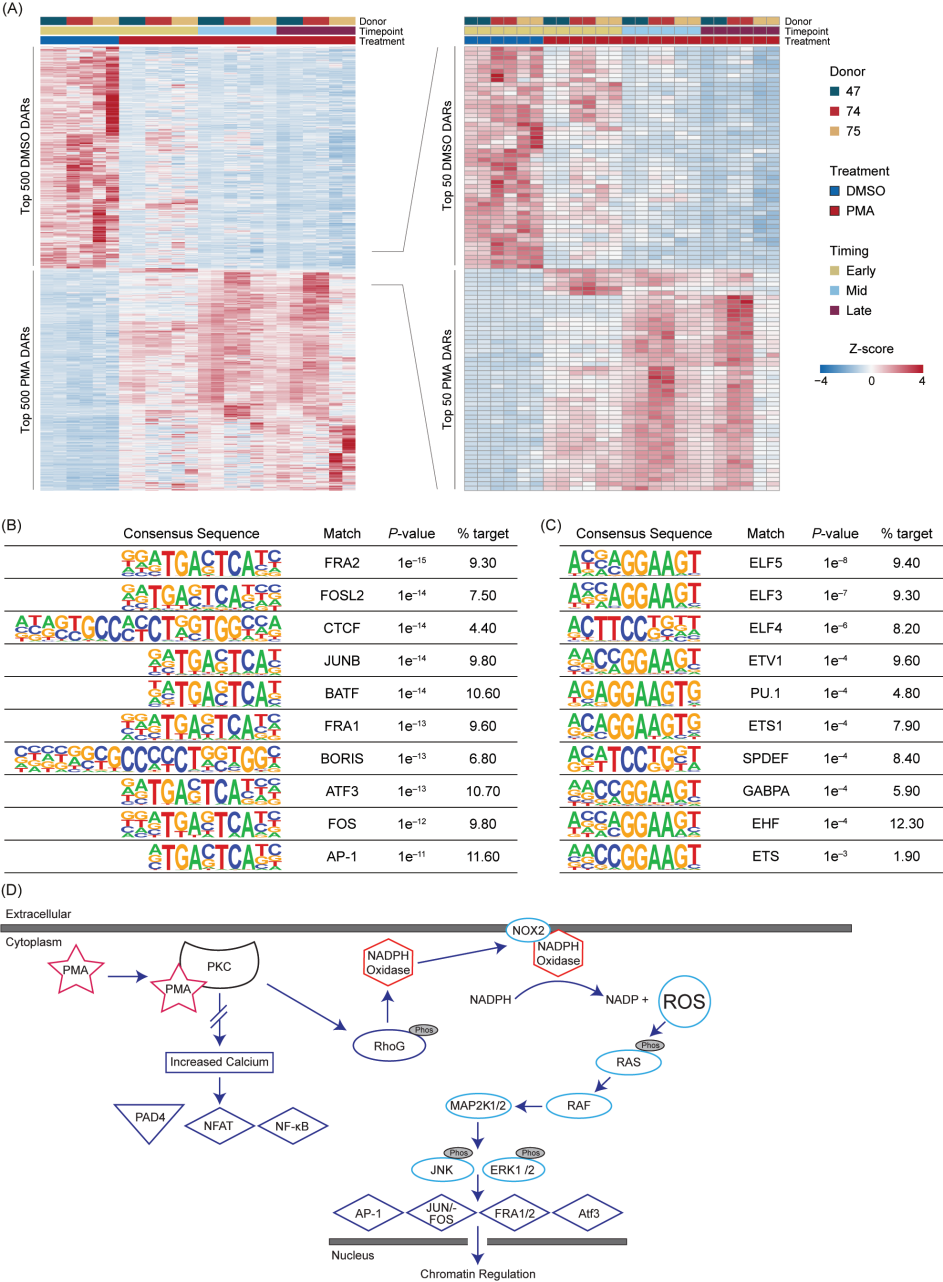
PMA-induced NET formation leads to increased accessibility in RAS-associated transcription factors. **(A)** Z-score normalized heatmap representation of the top 500 (left panel) differential accessible regions (DARs) for each Early dimethyl sulfoxide (DMSO) and phorbol 12-myristate 13-acetate (PMA) based on the Wald score [DESeq2 p.adj >0.01 and log_2_(fold change) less than −1.5 or greater than 1.5]. The top 100 DARs (right panel) were then filtered by the Wald score (50 upregulated in DMSO and 50 upregulated in PMA). Donors are indicated by color (D47, blue; D74, red; D75, gold). Timepoints (Early, Mid, and Late) are graphed per donor. Treatment is indicated by DMSO (blue) and PMA (red). Columns are organized by treatment, timepoint, and then donor across all conditions, and rows are hierarchically clustered. **(B)** HOMER analysis from the top 1,000 upregulated DARs in PMA samples [DESeq2 p.adj > 0.01 and log2(fold change) >1.5]. **(C)** Similar to **Figure 4B** but upregulated in DMSO samples [log_2_(fold change) <1.5]. **(D)** A potential mechanism for transcription factor (TF) activation through PMA-activated NET formation. Briefly, PMA activates PKC, which causes activation of NADPH and NOX through RhoG to generate an increase in intracellular reactive oxygen species (ROS). ROS then activate the RAS/RAF pathway and lead to the downstream activation of TFs such as AP-1, JUN/FOS, FRA1, FRA2, and ATF3. There is no increased calcium level caused by PKC activation; therefore, PAD4, NFAT, and NF-κB are not activated.

To obtain the highest significant DARs based on their Wald test score, the top 15% (500) upregulated in Early DMSO and the top 500 DARs for all PMA timepoints were selected (**Supplementary Table 4**). The DARs were then z-score normalized and ordered based on treatment and time (**Figure 4A**). There was some variability in the PMA DARs, with the largest differences being between Early PMA and Mid PMA. The data were further reduced to the top 1.5% DARs (50) in DMSO or PMA to highlight the largest differences between the treatments and timepoints. There were numerous DARs that appeared Early and then were lost as well as the opposite of a lack of signal increasing into the Mid timepoint (**Figure 4A** – right). These data also support the similarities between the Mid and Late PMA treatments.

The top 1,000 DARs from DMSO or PMA were then used for HOMER analysis (**Figures 4B, C**). In the PMA DARs, TFs previously associated with NET formation or inflammation, such as FRA1 (38), FRA2 (18), JUN/FOS (18), ATF3 (39), and AP-1 (18), were among the more prevalent (**Figures 4B, D**). CTCF was also present in 4.4% of the target sequences of the PMA DARs, which indicated a potential role of CTCF in the regulation of the chromatin response; however, CTCF is often overrepresented in HOMER analysis (40), therefore future studies should probe the potential impact of CTCF in chromatin regulation during NET formation. In DMSO DARs, there was not a strong correlation to NET-formation-associated TF binding sites or many direct pathways (**Figure 4C**). However, one of the top HOMER results was from the ELF family, which has been associated with autoimmune disease, and the reduction in ELF4 in macrophages has been correlated to increased neutrophilic infiltration (41, 42).

Overall, TF binding sites associated with PAD4, such as NF-AT and NF-κb, were not well represented in upregulated or downregulated DARs (**Figures 4B, C**), which could be modulated by a lack of intracellular calcium or other mechanistic reasons (**Figure 4D**). The representation of RAS-associated TFs seen in **Figure 4B** support previous findings of RAS activation through PMA stimulation through NOX dependency (18, 43). These results highlight the importance of these TFs and their potential roles in chromatin regulation during NET formation (41).

## Discussion

NET formation is a key component of the innate immune response, playing a crucial role in trapping and neutralizing pathogens. However, this process can become dysregulated and lead to sepsis and immunothrombotic diseases (44, 45). The transition from a beneficial to a pathogenic response is not well understood but may be driven by an excess of pro-inflammatory cytokines, inefficiencies in inhibitory molecules such as IL-10 (46), or repressive mechanisms. Additionally, the release of NET components, particularly chromatin, can contribute to cytotoxicity, exacerbating the pathological state. Therapeutics targeting NET formation have been challenging and not widely approved in clinical settings (47), indicating that a deeper understanding of the underlying mechanisms is likely needed to develop effective treatments. The molecular processes occurring during NET formation might vary significantly under (patho)physiological conditions and thus deciphering the molecular choreography underlying NET formation could provide important insights into these mechanisms governing both host defense and inflammation-associated pathology. Changes in chromatin architecture are an important early step in altering gene expression and initiating cellular regulatory programs, likely dictating the fate of neutrophils and shaping the ensuing immune response.

We used PMA to study chromatin accessibility during NET formation due to its robust and consistent induction of NETs. Unlike lipopolysaccharide (LPS) or other more physiologically relevant stimuli, PMA directly activates protein kinase C (PKC), leading to a highly reproducible and synchronous activation of downstream signaling pathways, including the RAS/RAF/MEK/ERK cascade, which are crucial for some types of NET formation (6). This consistency is important in generating uniformity in cell activation to ensure the observed changes in chromatin accessibility were due to the induced biological process rather than variability in stimulus response. PMA-induced NET formation involves the production of ROS and other signaling molecules, providing a well-characterized model to dissect the molecular mechanisms underlying chromatin remodeling. Furthermore, as little is known about how chromatin structure is changed during the early stages of NET formation, we chose to perform a focused characterization of chromatin changes, which can be utilized to interpret the bulk of the existing literature using PMA induction. Future studies will investigate whether the chromatin accessibility changes described here are specific to PMA-induced NET formation or are common to other NET inducing stimuli.

We have previously shown NET formation in isolated neutrophils with 100 nM PMA and have developed ATAC-Seq using the same stimulation conditions with an added formaldehyde fixation at specific timepoints to maintain chromatin structure within the nucleus and avoid NET release (**Supplementary Figure 3F**) (26). Although 100 nM PMA is a high concentration in isolated conditions, we wanted to ensure a rapid response of the entire population of cells to ensure uniform results during ATAC-Seq. Overall, we noticed an increase in noise and a lack of overlap of peaks being called between the standard and fixed ATAC-Seq samples (**Supplementary Figures 1B, C**). We have found that accessible chromatin regions in fixed samples were consistent across multiple donors, similar to other primary immune cell types (28, 48). We observed some donor-to-donor variability, but we generated a set of over 22,000 consensus peaks that were shared among 50% of the donors tested.

PMA is a strong inducer of NET formation through the activation of PKC, which leads to increased intracellular ROS through NADPH oxidase (NOX) (18, 19). We used our ATAC-Seq method to study PMA-induced neutrophils, first showing that DMSO-treated neutrophils were similar to untreated samples for up to 90 min post-treatment (**Figure 2B**). When neutrophils were treated with PMA, there was a consistent response across donors at multiple timepoints, suggesting an ordered and mechanistic regulatory program. A unique change in fragment profile could be seen as early as 30 min (**Figure 2D**), suggesting that chromatin re-organization happens before NET production, which can take several hours following PMA stimulation (26).

In concert with this early fragmentation change, we identified approximately 1,500 DARs that occurred between PMA- and DMSO-treated cells at 30 min, and the majority of these remained so up to 90 min. Globally, PMA-treated samples clustered away from DMSO-treated samples in an unsupervised clustering analysis (PCA), with sub-clustering by timepoint among the PMA-treated samples and limited donor effects. Protein Kinase C - β (*PRKCB*) has previously been shown to be upregulated during PMA stimulation (19, 49) and showed a stable increase in chromatin accessibility. More complex chromatin accessibility changes across the time course were evident at loci such as *ACTG1*, which has been shown to have an initial burst of transcription at 30 min followed by a reduction at 60 min in PMA-induced neutrophils (6), which corresponded to more accessible chromatin at 30 min followed by a reduction in accessibility by 60 min (**Figure 3B**).

Interestingly, we observed a gain of accessibility at DARs containing binding sites for the transcription factors JUN/FOS, FRA1, FRA2, ATF3, and AP-1 (18, 38, 39, 49–51) (**Figure 4B**). Upon PMA stimulation, an increase of ROS intracellularly can lead to various effects including the activation of the RAS pathway and activation of these same TFs, but not other TFs such as PAD4, NF-AT, or NF-kB (18, 19, 38, 52). There was not a strong signal for TFs associated with reduced chromatin accessibility, with the most significant hits occurring in the ELF TF family, which is known to be involved with many processes, including cell proliferation (53). A potential reason for a weaker signal in TFs associated with the loss of accessibility could be that the decondensation of the chromatin is targeted through a variety of TFs or another mechanism. The transcription factor motif findings support previous data that PMA-stimulated neutrophils act via a NOX-dependent mechanism, which activates the RAS pathway (**Figure 4D**), as opposed to when NET formation is activated through the NOX-independent pathway, which leads to the activation of PAD4 and subsequently NFAT and NF-kB (8, 19, 49). Importantly, we did not identify NFAT or NF-kB binding sites in our HOMER analysis of the top 1,000 DARs (**Figures 4B, C**).

Our findings deepen our comprehension of the epigenetic blueprint governing PMA-stimulated NET formation, and understanding the changes in chromatin accessibility may hold significant clinical implications, potentially leading to more targeted therapies. Such organized changes could reveal consistent epigenetic modifications and transcriptional programs essential for NET formation and immune response, highlighting potential therapeutic targets for diseases characterized by aberrant NET formation. The targets could lead to the development of drugs aimed at specific genes involved in pathological NET formation or the modulation of feedback loops. Additionally, understanding how PMA-induced chromatin changes compare with those triggered by more physiologically relevant stimuli, such as LPS or naturally occurring cytokines, could provide insights into the broader landscape of neutrophil epigenetics and the molecular mechanisms underlying NET formation. This knowledge can further elucidate how various stimuli influence physiological epigenetic processes, such as differentiation, activation, and response to pathogens, to offer a comprehensive view of neutrophil function in health and disease.

## Data Availability

The original contributions presented in the study are publicly available. This data can be found here: https://www.ncbi.nlm.nih.gov/bioproject/?term=PRJNA1120432.
