# Supplementary material for: Understanding the complex chromatin dynamics in primary human neutrophils during PMA-induced NET formation
Source: Front Immunol. 2024 Oct 25;15:1445638. doi: 10.3389/fimmu.2024.1445638 (PMC11544126; doi:10.3389/fimmu.2024.1445638)
Supplement: Supplementary file 1 [file DataSheet1.docx]

Supplementary Material

# Figures

**Supplementary Figure 1:** **(A)** Total number of merged mapped reads for the samples. The donors are indicated on the x-axis, and the number of mapped reads (in millions) is on the y-axis. **(B)** A comparison of FRiP (x-axis) with the total number of MACS2 peaks (q < 0.01) for each sample (merged replicates) including D32. Fixed samples are indicated by circles, and the unfixed sample is indicated by a triangle. Each donor number is indicated, and the dashed line represents y=x. Correlation (R²) was 0.60. **(C)** Similar to Figure 1D but D32 (unfixed sample) was included.

**Supplementary Figure 2:** **(A)** Similar to Supplementary Figure 1A but using the merged treated samples. **(B)** Similar to Figure 1C but using the merged treated samples. **(E)** A comparison of Fraction of Reads in Peaks (FRiP) (x-axis) with the total number of MACS2 peaks (q < 0.01) for each sample (merged replicates). DMSO samples are indicated by squares and PMA samples are indicated by circles. The timepoint is represented by color (Early – gold, Mid – blue, Late – purple). Each donor number is indicated, and the dashed line represents y=x. Correlation (R²) was 0.76.

**Supplementary Figure 3:** **(A)** 100 nM PMA and DMSO vehicle control on isolated neutrophils (D73) using Cytotox green dye and S3 Incucyte imaging system. **(B)** Representative stimulation images of isolated primary neutrophils (D47) at T=3 hours with either 100 nM phorbol 12-myristate 13-acetate (PMA) or DMSO. Staining was completed with DAPI, anti-H3.1 (Volition, ABV0003), membrane dye (Thermo, C10045), and then merged images. Scale Bars, 20 µm. **(C)** Similar to (A) but anti-H3R8cit (Abcam, ab232939) was used instead of anti-H3.1. Scale Bars, 20 µm. **(D)** Similar to (A) but anti-MPO (Abcam, ab221847) was used instead of anti-H3.1. Scale Bars, 20 µm. **(E)** Similar to (A) but anti-Neutrophil Elastase (NE) (Thermo, MA5-32548) was used instead of anti-H3.1. Scale Bars, 20 µm. **(F)** Similar to (A) but anti-nucleosome (Volition, ABV0002) was used instead of anti-H3.1. Scale Bars, 20 µM.

1. **Tables**

Supplementary Tables.xlsx: (Table 1) Donor information for all donors in the study. (Table 2) Antibodies used for immunocytochemistry. (Table 3) Illumina Sequencing statistics of all samples. (Table 4) Top 500 upregulated differentially accessible regions (DARs) and the Top 500 downregulated DARs based on the Wald test.
